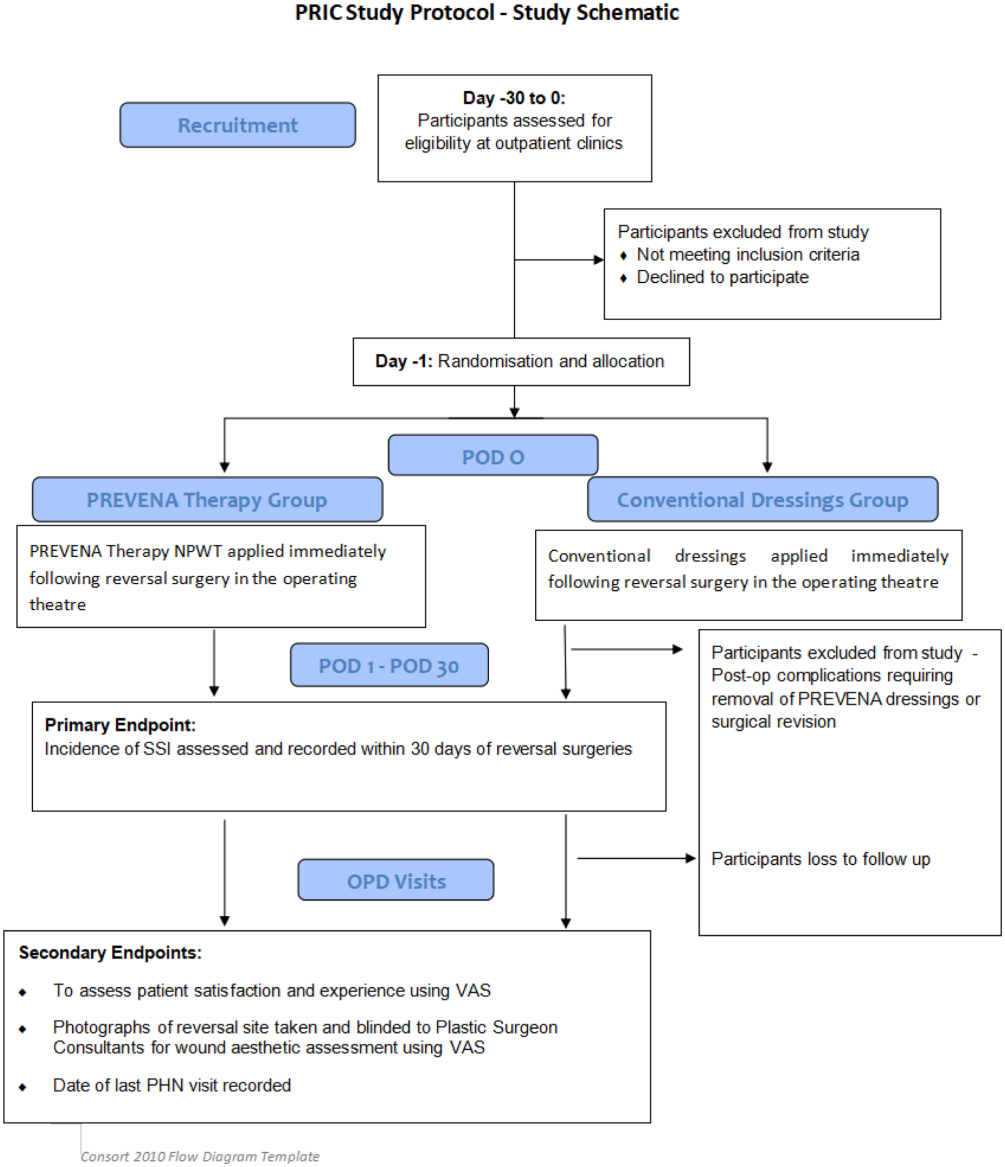# Application of PREVENA (Surgical Incision Protection System) in reducing surgical site infections following reversal of ileostomy or colostomy: the PRIC study protocol

**DOI:** 10.1007/s00384-022-04153-3

**Published:** 2022-04-29

**Authors:** Ernest Z. Low, Timothy S. Nugent, Niall J. O’Sullivan, Dara Kavanagh, John O. Larkin, Paul H. McCormick, Brian J. Mehigan, Michael E. Kelly

**Affiliations:** 1 Department of Colorectal Surgery, St James’s Hospital, Trinity College Dublin, Dublin, Ireland; 2 Department of Colorectal Surgery, Tallaght University Hospital, Dublin, Ireland

**Keywords:** Reversal surgery, Ileostomy, Colostomy, Colorectal surgery, Negative pressure dressings, Surgical site infection, PREVENA therapy

## Abstract

**Aim:**

There is a current lack of evidence in the literature to support the routine use of negative pressure wound therapy (NPWT) to reduce the risk of surgical site infections (SSI) in the setting of ileostomy or colostomy reversal. The aim of this study is to examine whether routine NPWT confers a lower rate of SSI than conventional dressings following reversal of ileostomy or colostomy.

**Methods:**

The PRIC study is a randomized, controlled, open-label, multi-centre superiority trial to assess whether routine NPWT following wound closure confers a lower rate of SSI following reversal of ileostomy or colostomy when compared to conventional dressings. Participants will be consecutively identified and recruited. Eligible participants will be randomized in a 1:1 allocation ratio, to receive either the NPWT (PREVENA) dressings or conventional dressings which will be applied immediately upon completion of surgery. PREVENA dressings will remain applied for a duration of 7 days. Surgical wounds will then be examined on post-operative day seven as well as during follow-up appointments in OPD for any evidence of SSI. In the interim, public health nurses (PHN) will provide out-patient support services incorporating wound assessment and care as part of a routine basis. Study investigators will liaise with PHN to gather the relevant data in relation to the time to wound healing. Our primary endpoint is the incidence of SSI within 30 days of stoma reversal. Secondary endpoints include measuring time to wound healing, evaluating wound healing and aesthetics and assessing patient satisfaction.

**Conclusion:**

The PRIC study will assess whether routine NPWT following wound closure is superior to conventional dressings in the reduction of SSI following reversal of ileostomy or colostomy and ascertain whether routine NPWT should be considered the new standard of care.

## Introduction

Surgical site infections (SSI) remain one of the most common complications following reversal of ileostomy and colostomy [1, 2]. They contribute to a longer length of stay and increased healthcare expenditure and pose adverse psychological effects on patients [3, 4]. The use of various skin closure techniques has been studied with the aim of reducing SSI, for example, the purse-string closure method has been reported to have considerable success [5, 6]. Similarly, the use of routine negative pressure wound therapy (NPWT) has been postulated as an alternative method to mitigate the risk of developing SSI [7–11].

PREVENA Therapy is a form of incisional negative pressure wound therapy (I-NPWT) that has been widely used in the management of closed surgical incisions. PREVENA Incision Management System is a type of disposable, customizable and powered negative pressure system designed to help manage and protect surgical incisions and their surrounding environment. These dressings are designed to hold incision edges together, as well as remove both fluids and infectious agents. They act as a barrier to external contamination by delivering a continuous negative pressure of 125 mmHg for up to 7 days [12]. Physiologically, they promote wound healing by increasing tissue granulation, promoting angiogenesis and increasing perfusion to tissue via fluid evacuation [13].

In recent years, there have been multiple studies comparing the use of PREVENA Therapy to conventional dressings in the management of surgical wounds, particularly in the setting of vascular surgeries, post-caesarean infections and colorectal resections [14–19]. To date, there is a lack of evidence to support the routine use of NPWT or PREVENA Therapy in the setting of stoma reversal. Patients undergoing elective reversal of ileostomy or colostomy are deemed vulnerable to developing SSI. In view of the significant patient morbidity and healthcare burden associated with SSI as well as the potential impact of PREVENA Therapy in reducing these complications, this study aims to assess whether routine NPWT is superior to conventional dressings in the prevention of SSI.

## Methods

PRIC study is registered on the ClinicalTrials.gov platform—NCT0497493. Approval by the Research Ethics Committee at St. James’s Hospital/Tallaght University Hospital has been granted.

### Study setting and design

This study is designed as a randomized, controlled, open-label, multi-centre superiority trial with two parallel groups with a primary endpoint of measuring SSI incidence within 30 days of reversal of ileostomy or colostomy. The trial will be conducted at two centres located in Dublin, Ireland: St James’s Hospital and Tallaght University Hospital.

### Objectives

The primary aim of PRIC study is to examine whether routine NPWT (PREVENA) confers a lower rate of surgical site infections than conventional dressings following reversal of loop ileostomy or colostomy. The secondary objectives of PRIC study are:

1. To assess if NPWT confers a shorter time to wound healing
2. To assess patient experience and satisfaction with NPWT and conventional dressings
3. To assess wound aesthetics

### Inclusion criteria

PRIC Study will include patients who are electively admitted to St. James’s Hospital or Tallaght University Hospital for reversal of loop ileostomy or colostomy. Patients must be 16 years of age or older and consent to the follow-up protocols.

### Exclusion criteria

Patients will be excluded from the PRIC Study if they are less than 16 years of age at screening or if they fail to attend for their regular OPD follow-up appointments. Patients will also be excluded and recorded as treatment failures if dressings, either PREVENA or conventional, are removed outside of their defined time periods.

### Patient identification and consent

Patients undergoing reversal surgeries will be assessed in outpatient clinics in SJH and TUH prior to admission for these elective procedures. Eligible participants will be identified and recruited. Explanation regarding PRIC study as well as a patient information leaflet will be provided during these visits, and eligible participants will have a period of time prior to elective admission to consider their participation. All patient participants must provide written informed consent to enter this study. The PRIC Study enrolment will commence on the 1st of May 2022 at both SJH and TUH.

### Interventions

Following selection and attainment of informed consent, all participants will be randomly assigned to either the PREVENA therapy group or conventional dressings group with a 1:1 allocation ratio via stratified randomisation. Participants will be stratified by their baseline comorbidity state and will be stratified into one of the three groups, namely:

i) No diabetes nor immunosuppressant use
ii) Diabetic patients (both insulin, non-insulin dependent, type one or type two)
iii) Use of immunosuppressants

Following stratification, participants will be systematically randomized in a 1:1 allocation ratio into either the PREVENA group or the conventional dressing group. The conventional dressings that are used in the study will be non-antimicrobial, standard surgical dressing. On the day of elective admission, the relevant primary or co-investigators will perform this randomisation by picking one of the sealed envelopes containing the assignment. There will be equal proportion of assignments to either group (PREVENA and conventional dressings) to achieve a 1:1 allocation ratio. The conventional dressings that are used in the study will be non-antimicrobial, standard adherent surgical dressing. Due to the nature of the intervention, neither the participants nor the staff can be blinded to allocation. Prophylactic antibiotics will be administered following reversal surgeries as per local guidelines. Initial dose will be administered during induction of the surgeries, followed by three more doses administered post-operatively. Wound closure will be performed in a standardized manner, and the randomized dressing will be applied immediately after wound closure. The PREVENA dressings will be left in situ for 7 days at which point the wound will be inspected for any evidence of SSI. Wound swabs will be performed for culture and sensitivity for any incidence or suspicion of SSI.

Criteria for discontinuing PREVENA Therapy include post-operative complications requiring removal of PREVENA dressings or requiring surgical re-intervention. A number of possible risks associated with the use of negative pressure dressing PREVENA will be monitored which include but are not limited to discomfort with application or re-application, skin irritation or reaction to dressing materials used, wound bleeding, infection and retained dressing material. Surgical site wounds will be closely monitored, and should any of these occurrences arise, they will be promptly managed and treated. We acknowledge and permit certain relevant concomitant care such as the use of nutritional supplements. Nutritional supplements will be permitted to participants if deemed beneficial or necessary by a dietitian.

### Outcomes

The primary outcome of PRIC Study is to measure the incidence of investigator assessed SSI within 30 days following reversal of ileostomy or colostomy (confirmed with culture and sensitivity microbial growths). The secondary outcomes are to assess patient satisfaction and experience with either dressing via a visual analogue score (VAS), to measure the time to wound healing and to evaluate wound healing during routine OPD visits through taking photographs and VAS rating by our plastic consultant colleagues.

### Sample size

Based on published literature, we hypothesize a 60% reduction in SSI rates following the routine use of NPWT (25 to 10% at closure site). The study will be a randomized, controlled, open-label trial with two parallel arms comparing standard and routine NPWT applied immediately after reversal of ileostomy or colostomy. The sample size required to achieve a power of 1-β = 0.80 and at the level α = 0.05 under these assumptions (including a margin error with application failure, saturation and patient non-compliance) amounts to 100 patients needed for each arm; thus 2 × 100 = 200 participants are required. We incorporate a 10% dropout rate in our sample size calculation, and this would inflate the sample size to approximately 222 participants.

## Data collection and management

SSI is assessed using the definition criteria established by the ‘United States Centers for Disease Control and Prevention’ [20]. Post-operatively, the study investigators will assess for any evidence of SSI. Following discharge from the study sites, PHN will routinely carry out home support services including wound assessment and wound care for all participants who have underwent reversal surgery. Should any concerns regarding SSI arise, patients will be referred back to study sites and be reassessed for wound SSI by study investigators. At this point SSI will be confirmed with culture and sensitivity microbial growths. If there are no such concerns of SSI during the house visits, all participants will be routinely reviewed by study investigators for any signs of SSI during their routine OPD visits. The follow-up OPD visits will typically take place approximately 30 days following discharge from the study sites. To measure the time to wound healing, the investigators will liaise with the PHN to gather the relevant data on the time to wound healing, which is calculated from the day of reversal surgery to the day of last PHN home visit.

To assess patient satisfaction, the investigators will be applying a visual analogue scale (VAS) as the measurement instrument, and patient participants will be invited to complete this during OPD visits. The investigators will also take photographs of the surgical site of reversals during routine OPD visits, and these photographs will be blinded to assess for wound healing. Participant retention is strongly promoted through strict scheduling of follow-up appointments.

Data will be collected at both study sites, SJH and TUH, using hospital network computer. They will be password protected and stored in an encrypted folder. To safeguard confidentiality, data will be pseudo-anonymised immediately at the point of collection, and the key code that links the data to patients will be a hard-copy document kept in a secure cabinet on the study site and will be irrevocably destroyed once the study period has been completed. All reports used by the data coordinating centre will be prepared in a manner such that no individual subject can be identified. All data controllers have completed the General Data Protection Regulation (GDPR) training and have experience in collecting and handling patient data from previous studies.

### Data analysis

Data analysis, processing and calculation of inferential statistics will be performed primarily using SPSS software. In relation to primary endpoint analysis, which assesses the presence of SSI within 30 days following reversal surgeries, a Chi-squared test will be used to compare the SSI incidence rates between the two randomized groups (PREVENA vs conventional surgical dressings). The time to wound healing, patient experience and satisfaction and the degree of wound healing and wound aesthetics will be presented as secondary outcomes. A t-Test will be applied to compare the time to wound healing between the randomized groups as appropriate. Similarly, t-Test will be applied to compare the level of satisfaction between patients of the randomized groups. All outcome measures will be presented with 95% confidence intervals and two-sided test *p* values.

### Subgroup analysis

We intend to conduct a subgroup analysis given the strong biological rationale and potential interaction effects these variables may have on measured outcomes. These variables include age, presence of co-morbidities such as diabetes mellitus and the use of immunosuppressants which may impair the process of wound healing.

### Data monitoring

An interim analysis will be conducted at month 6 to assess recruitment, the overall SSI rate between two groups and the failure of protocol rates. The PRIC Study may potentially be expanded internationally and conducted at other clinical facilities. The data monitoring process and interim analysis will be carried out independently from any third parties and any potential sponsors.

## Discussion

At present, there is a lack of consensus on the routine use of NPWT in the setting of stoma reversal. To date, there have been four randomized trials that have investigated the use of NPWT in stoma reversal, and the results from these studies are conflicting and inconclusive. The NEPTUNE trial observed that the prophylactic use of NPWT was not associated with a decrease in 30-day SSI incidence rate when compared to standard dressings, with the 30-day SSI incidence of the NPWT group being 32% versus 34% in the standard dressing group [17]. Uchino et al. also noted no difference in the efficacy rates between the use of NPWT and purse-string suture methods in preventing SSI [21]. Similarly, Carrano et al. reported similar incidence rates of SSI and wound complications in both the NPWT and conventional dressing cohorts [22]. In contrast, the trial conducted by Wierdak et al. reported a positive benefit in the use of prophylactic NPWT in the setting of ileostomy closure, with a reduced incidence of SSI and wound healing complications. They had a SSI incidence of 5.7% in the NPWT group versus 22.2% in the standard dressing cohort [23]. Similarly, wound healing complications, defined as any condition of the wound that required post-operative intervention other than a change of dressing or removal of stiches, were significantly lower in the NPWT group (8.6% versus 30.6%) [23]. In addition to these conflicting results, these studies are not sufficiently powered, thereby supporting the need for further evaluation and support the relevance of PRIC Study in establishing whether the routine use of NPWT does reduce SSI rate when compared to using conventional dressings.

In addition, current literature lacks research studies that employ microbiology wound swabs and culture in their assessment of SSI. PRIC Study will involve the use of microbiology wound swab, culture and sensitivity to help improve the accuracy of assessing and reporting SSI incidence. And to our knowledge, the PRIC Study is the first study that employs microbiological wound swabs in assessing SSI in the setting of reversal of ileostomy and colostomy surgeries.

In relation to the indirect or non-medical standpoint, the financial costs associated with the incidence of SSI are substantial. SSI is thought to contribute to twice as much as the inpatient costs than a patient without SSI [24]. They contribute to an increased financial burden via prolonged hospitalization, reoperation/intervention and/or readmission [4]. The PRIC Study will assess not only the incidence of SSI, but also the time required to wound healing, and these results may be used to inform future cost–benefit analytical studies.

Although the purse-string closure technique has been reported to have considerable success in reducing the rate of SSI incidence [5, 6], there are concerns pertaining to this method primarily due to the slow time to complete wound healing and unsatisfactory wound aesthetics [25]. The PRIC Study will also evaluate whether the use of PREVENA Therapy leads to better wound healing and appearance as compared to conventional dressings. Whilst it is crucial that we continue to seek better strategies to reduce the incidence of SSI and other associated post-operative complications, it is equally important that we do not neglect the psychological aspects and patients’ experience with any proposed interventions. Stoma reversals are known to incur prolonged social and psychological impacts on patients [26] despite their low mortality rates of less than 4% [27]. Following stoma reversals, patients are at risk of developing post-operative complications such as small bowel obstruction, SSI, parastomal hernia, anastomotic leak and enterocutaneous fistulae [28] and often have to cope with significant alteration in bowel function which greatly affects daily routines [29]. The PRIC Study will assess the patient experience with their application of PREVENA Therapy and will address the concerns and reservations associated with the use of PREVENA Therapy.